# 血清NSE、ProGRP和LDH在小细胞肺癌诊断治疗中的作用

**DOI:** 10.3779/j.issn.1009-3419.2016.09.05

**Published:** 2016-09-20

**Authors:** 彦 彭, 燕 王, 峻岭 李, 学志 郝, 兴胜 胡

**Affiliations:** 1 100021 北京，北京市朝阳区三环肿瘤医院肿瘤内科 Department of Medical Oncology, Beijing Chaoyang Sanhuan Cancer Hospital, Beijing 100021, China; 2 100021 北京，中国医学科学院肿瘤医院肿瘤内科 Department of Medical Oncology, Cancer Hospital Chinese Academy of Medical Sciences, Beijing 100021, China

**Keywords:** 小细胞肺癌, 肿瘤标志物, 神经元特异性烯醇化酶, 胃泌素释放肽前体, 乳酸脱氢酶, 疗效, 复发, Small cell lung cancer, Tumor marker, Neuron specific enolase, Pro-gastrin-releasing peptide, Lactic dehydrogenase, Efficacy, Relapse

## Abstract

**背景与目的:**

小细胞肺癌（small cell lung cancer, SCLC）是一种生长迅速、具有神经内分泌特性的肿瘤。血清神经元特异性烯醇化酶（neuron specific enolase, NSE）、胃泌素释放肽前体（pro-gastrin-releasing peptide, ProGRP）和乳酸脱氢酶（lactic dehydrogenase, LDH）已在SCLC的诊断和治疗中起到一定的辅助作用，本研究旨在通过治疗前后SCLC患者NSE、ProGRP和LDH的变化探讨标志物在肿瘤分期、疗效评价及预测复发方面的价值。

**方法:**

纳入中国医学科学院肿瘤医院的SCLC初治病例，回顾性分析其临床数据，包括临床特征、治疗前及2周期化疗后的血清NSE、ProGRP及LDH，疗效及无进展生存期。

**结果:**

治疗前，广泛期（extensive disease, ED）患者NSE、ProGRP及LDH均高于局限期（limited disease, LD）（*P* < 0.005）；LD患者的NSE水平随淋巴结分期的升高而明显增加（*P*=0.010）；有体重下降的患者NSE及LDH均高于无体重下降者（*P*=0.032, *P*=0.014）。化疗2周期后，有效患者的NSE及ProGRP下降程度明显高于疗效为稳定或无效的患者（*P*=0.015, *P*=0.002）。LD组化疗周期数 > 4个及治疗后ProGRP下降明显的患者较化疗周期数≤4个及ProGRP下降不明显的患者复发风险低；而远处转移数目≤2个、疗前LDH正常及治疗后ProGRP的明显下降，提示ED患者的近期复发风险低。此外，肿瘤复发类型（敏感复发、耐药复发、难治复发）与化疗后ProGRP下降程度呈负相关（*P*=0.044）。多因素分析结果提示治疗周期数是LD组SCLC近期复发的独立影响因素，远处转移数目及治疗后ProGRP的下降程度是ED组SCLC近期复发的独立影响因素。

**结论:**

血清肿瘤标志物升高的程度与肿瘤负荷相关，ProGRP在治疗后的下降程度可能预测疗效及复发风险。

## Abstract (en)

**Background and objective:**

Small cell lung cancer (SCLC) is a rapidly growing tumor with characteristic of neuroendocrine cellular function. Neuron specific enolase (NSE), pro-gastrin-releasing peptide (ProGRP) and lactic dehydrogenase (LDH) are valuable in diagnosis and treatment of SCLC. By analyzing the variation of NSE, ProGRP and LDH before and after treatment, the aim of this study is to investigate the efficacy of tumor markers in diagnostic staging, therapeutic evaluation and prediction of disease relapsing.

**Methods:**

Patients with SCLC who receiving the first line chemotherapy in Cancer Hospital, Chinese Academy of Medical Sciences were enrolled and retrospectively analyzed. Clinical characteristic (includes NSE, ProGRP and LDH level before and after 2 cycles chemotherapy), efficacy evaluation, progression-free survival (PFS) were analyzed.

**Results:**

Before treatment, Serum NSE, ProGRP and LDH in patients with extensive disease (ED) were significantly higher than those with limited disease (LD)(all *P* < 0.005); NSE level increased obviously accompanied by increase of lymph nodes stage in LD group (*P*=0.010); Patients with weight reduction when diagnosis had higher NSE and LDH than those without loss of weight (*P*=0.032, *P*=0.014). After 2 cycles chemotherapy, decrease of NSE and ProGRP in effective group was higher than which in stable and ineffective groups (*P*=0.015, *P*=0.002). The relapse risk was lower in patients who accepted > 4 cycles chemotherapy and with obvious decrease of ProGRP than those who accepted ≤4 cycles chemotherapy and with less obvious decrease of ProGRP in LD group; ED patients with no more than 2 distant metastasis, normal LDH level before treatment and obvious decrease of ProGRP after chemotherapy had lower short term relapse risk. In addition, the types of relapse (sensitive relapse, drug resistance relapse and refractory relapse) were negatively correlated with decrease of ProGRP (*P*=0.044). By multivariate analysis, numbers of chemotherapy cycle was independent prognostic factor for PFS in LD SCLC; numbers of distant metastasis and decrease of ProGRP were independent prognostic factors for PFS in ED SCLC.

**Conclusion:**

Increase level of serum tumor markers is related to tumor burden. Decrease level of ProGRP after treatment may prognose efficacy and relapse risk.

肺癌是目前我国乃至全世界发病率与死亡率最高的恶性肿瘤。根据生物学行为的不同，将肺癌分为非小细胞肺癌（non-small cell lung cancer, NSCLC）和小细胞肺癌（small cell lung cancer, SCLC）。SCLC约占肺癌的20%，肿瘤恶性程度高，诊断时约2/3有远处转移，主要的治疗手段为化疗及放疗。尽管SCLC对化疗及放疗较敏感，但肿瘤复发较快，及早判断疗效，预期生存对指导临床治疗及减轻患者痛苦有极大帮助。肿瘤标志物在SCLC的疗效评价、监测复发及预后判断方面有着重要的作用^[1]^。血清神经元特异性烯醇化酶（neuron specific enolase, NSE）及胃泌素释放肽前体（pro-gastrin-releasing peptide, ProGRP）在SCLC中高表达，联合应用提高了诊断的敏感性^[2]^。二者在疗效评价及预测复发方面资料相对较少。既往研究提示乳酸脱氢酶（lactic dehydrogenase, LDH）水平是SCLC的预后因素^[3]^。本研究分析中国医学科学院肿瘤医院治疗的SCLC的数据，探讨NSE、ProGRP及LDH在疗前分期、疗效评价及预测复发中的作用。

## 1 资料与方法

### 1.1 一般资料

收集自2013年4月-2014年12月在中国医学科学院肿瘤医院治疗的SCLC初诊患者139例。所有患者经病理或细胞学以及免疫组化确诊为SCLC。

### 1.2 治疗方案

所有患者均接受一线化疗，化疗方案的选择由临床医师按照美国国立综合癌症网络治疗指南并根据患者一般状况选择。根据分期的不同部分患者进行了同步或序贯胸部放疗。

### 1.3 临床特征

通过查阅病历获得患者的临床基线特征及治疗数据。包括：患者性别、年龄、发病时体重变化、诊断时分期、一线化疗方案及其疗效、放疗方式和一线治疗后疾病复发时间。

### 1.4 疗效评价及结果判定

按照实体瘤疗效评价标准（Response Evaluation Criteria in Solid Tumors, RECIST）1.1版^[4]^，分为完全缓解（complete response, CR）、部分缓解（partial response, PR）、疾病稳定（stable disease, SD）和疾病进展（progressive disease, PD）。无进展生存期（progression-free survival, PFS）为自首次化疗至首次PD或死亡时间。将化疗结束后至复发时间小于3个月定义为难治性复发，3个月-6个月定义为耐药复发，6个月以上定义为敏感复发。血清指标检测方法：NSE为免疫电化学发光方法；ProGRP为免疫化学发光方法；LDH为酶法。正常值范围：NSE为0-18 ng/mL，ProGRP为0-50 pg/mL，LDH为135 U/L-225 U/L。中位随访时间为15个月。

### 1.5 统计学方法

采用SPSS 22.0统计学软件进行数据的统计学处理。因标志物数据不符合正态分布，计量资料采用中位数及四分位数间距描述，计数资料采用例数及百分比描述。标志物与临床因素间的关系比较采用秩和检验，预后因素分析采用*Cox*回归分析。*P* < 0.05为差异有统计学意义。

## 2 结果

### 2.1 患者基线特征

共纳入139例患者，其中男性103例（74.1%），女性36例（25.9%）；年龄中位60岁（32岁-81岁）。按照美国退伍军人肺癌协会分期方法分为局限期（limited disease, LD）和广泛期（extensive disease, ED）。本研究纳入LD患者86例（61.9%），ED患者53例（38.1%）。ED组患者的疗前NSE、ProGRP及LDH均高于LD组，差异有统计学意义（*P*值均 < 0.005）。诊断时有体重下降的患者NSE及LDH均高于无体重下降者（*P*=0.032, *P*=0.014）。LD患者的NSE水平随淋巴结分期的升高而明显增加（*P*=0.010）（表 1）。

**1:** 标志物与临床特征的关系（Md/Q） Correlation between tumor markers and clinical characteristic (Md/Q)

Group	NSE (ng/mL)	*Z*/*χ*^2^	*P*	ProGRP (pg/mL)	*Z*/*χ*^2^	*P*	LDH (U/L)	*Z*/*χ*^2^	*P*
Gender		-0.830	0.407		-1.383	0.167		-1.116	0.265
Male	33.92 (49.62)			518.94 (1, 579.27)			187.00 (68.00)		
Female	36.35 (25.27)			873.67 (1, 796.73)			191.00 (55.50)		
Age (yr)		-1.264	0.206		-0.265	0.791		-0.142	0.887
< 60	37.97 (45.87)			652.25 (1, 906.95)			190.00 (43.00)		
≥60	29.12 (35.20)			485.50 (1, 275.50)			187.00 (72.00)		
Stage		-6.049	< 0.001		-2.940	0.003		-4.510	< 0.001
LD	27.95 (19.01)			347.85 (848.80)			184.00 (39.00)		
ED	71.42 (69.03)			1, 030.19 (2, 690.15)			228.00 (157.00)		
Weight reduction		-2.140	0.032		-1.373	0.170		-2.464	0.014
No	32.31 (37.98)			445.53 (1, 347.23)			187.00 (51.50)		
Yes	43.39 (82.50)			1, 086.56 (2, 744.51)			218.00 (153.00)		
Lymph nodes stage in LD group		9.196	0.010		0.885	0.642		5.899	0.052
N1	26.30 (17.66)			279.26 (129.98)			199.00 (37.00)		
N2	28.11 (18.52)			429.11 (982.08)			183.00 (32.00)		
N3	47.42 (78.71)			355.71 (1, 009.53)			227.00 (92.50)		
LD: limited disease; ED: extensive disease; NSE: neuron specific enolase; ProGRP: pro-gastrin-releasing peptide; LDH: lactic dehydrogenase.									

### 2.2 治疗方案

全组患者完成化疗的中位数为6周期（2周期-8周期），化疗方案为依托泊苷联合顺铂75例（54.0%），依托泊苷联合卡铂58例（41.7%），ED组其他方案6例（4.3%），包括依托泊苷联合奈达铂4例，伊立替康联合顺铂2例。LD患者中有77例（89.5%）接受了胸部放疗，其中同步放疗46例（59.7%），序贯放疗31例（40.3%）；随着胸部放疗在广泛期SCLC治疗中作用的证实^[5]^，ED患者中有31例（58.5%）接受了胸部放疗，其中同步放疗3例（9.7%），序贯放疗28例（90.3%）。

### 2.3 缓解率

一线化疗中最好疗效为CR仅1例（0.7%），PR有120例（86.3%），SD为14例（10.1%），PD为4例（2.9%）。化疗2周期后NSE及ProGRP的浓度差异在各疗效组间有统计学意义，有效患者的NSE及ProGRP下降程度明显高于疗效为稳定或无效的患者（*P=*0.015, *P=*0.002）。ED组患者的NSE及LDH下降程度较LD组明显（*P* < 0.001, *P*=0.001）（表 2）。

**2:** 标志物下降程度与最好疗效、分期及复发类型的关系（Md/Q） Correlation between decrease of tumor markers (%) and the best efficacy and stageand three types of relapse (Md/Q)

Item	Decreaseof NSE (%)	*Z*/*χ*^2^	*P*	Decrease of ProGRP (%)	*Z*/*χ*^2^	*P*	Decrease of LDH (%)	*Z*/*χ*^2^	*P*
The best efficacy		8.386	0.015		12.906	0.002		0.837	0.658
CR+PR	68.00 (34.90)			77.10 (38.33)			14.11 (25.68)		
SD	53.05 (33.87)			23.51 (80.31)			9.68 (22.96)		
PD	44.45 (110.38)			6.53 (54.22)			16.97 (43.97)		
Stage		-3.569	< 0.001		-0.348	0.728		-3.430	0.001
LD	57.28 (36.83)			74.67 (35.84)			10.57 (24.65)		
ED	75.09 (30.26)			70.75 (73.56)			21.19 (34.89)		
Types of relapse		1.524	0.467		4.821	0.090		3.469	0.177
Sensitive relapse	68.55 (38.85)			81.77 (31.66)			9.68 (22.34)		
Drug resistance relapse	65.87 (32.87)			72.48 (62.14)			18.42 (15.09)		
Refractory relapse	74.10 (31.49)			58.31 (63.88)			19.92 (26.34)		
CR: complete response; PR: partial response; SD: stable disease; PD: progressive disease.									

### 2.4 近期复发

LD患者中位PFS为14个月，ED患者中位PFS为5个月。经过*Spearman*等级相关分析，随着敏感、耐药、难治复发类型的变化，ProGRP下降程度与复发类型呈负相关（*r*=-0.224, *P*=0.044）。单因素分析提示，在LD组中，化疗周期数 > 4个及治疗后ProGRP下降 > 70%的患者较化疗周期≤4个及ProGRP下降≤70%的患者复发风险低（*P*=0.007, *P*=0.037）；在ED组中，远处转移数目≤2个、疗前LDH正常及治疗后ProGRP的下降明显提示ED患者的近期复发风险低（*P*=0.007, *P*=0.036, *P*=0.010）（表 3）。多因素分析结果提示化疗周期数是局限期SCLC近期复发的影响因素；而远处转移数目及治疗后ProGRP的下降程度是广泛期SCLC近期复发的影响因素（*P*=0.031, *P*=0.014）。

**3:** LD组和ED组复发风险的单因素分析 Prognostic factors for PFS in LD and ED group by univariate analysis

Factors	LD			ED	
	*P*	HR (95%CI)		*P*	HR (95%CI)
Weight reduction (yes *vs* no)	0.583	1.252 (0.561-2.795)		0.402	1.319 (0.690-2.519)
Number of distant metastasis (> 2 *vs* ≤2)	-	-		0.007	3.208 (1.379-7.462)
Chemotherapy cycles (> 4 *vs* ≤4)	0.007	0.474 (0.276-0.817)		0.147	0.659 (0.375-1.157)
Thoracic radiotherapy (sequential *vs* concurrent)	0.080	1.663 (0.941-2.937)		-	-
Thoracic radiotherapy (yes *vs* no)	-	-		0.284	0.733 (0.415-1.293)
NSE (ng/mL) (> 18 *vs* ≤18)	0.499	1.256 (0.648-2.433)		0.257	2.294 (0.547-9.627)
ProGRP (pg/mL) (> 50 *vs* ≤50)	0.892	0.947 (0.429-2.090)		0.400	0.644 (0.230-1.798)
LDH (U/L) (> 225 *vs* ≤225)	0.866	1.071 (0.483-2.374)		0.036	1.889 (1.043-3.420)
Decrease of NSE (> 70% *vs* ≤70%)	0.538	1.196 (0.676-2.115)		0.354	1.319 (0.734-2.369)
Decrease of ProGRP (> 70% *vs* ≤70%)	0.037	0.523 (0.284-0.960)		0.010	0.427 (0.223-0.818)
Decrease of LDH (> 10% *vs* ≤10%)	0.160	1.515 (0.849-2.704)		0.816	1.081 (0.559-2.091)

## 3 讨论

SCLC具有神经内分泌功能，NSE是神经内分泌细胞特有的烯醇化酶的同工酶，在SCLC中有过量表达，阳性率为60%-80%^[6]^。胃泌素释放肽具有促胃泌素分泌作用，1994年Miyake等^[7]^开发出新的SCLC增殖因子ProGRP，在血液中较为稳定，用于评价SCLC的治疗疗效和早期复发。有研究^[8]^提示ED患者血清ProGRP水平高于LD患者且敏感性明显高于NSE。本项研究中ED组患者的疗前NSE、ProGRP及LDH均高于LD组，LD组中淋巴结转移分期为N3的患者NSE水平明显高于N2及N1，提示肿瘤标志物水平与肿瘤负荷及疾病分期有关。有研究提示诊断时体重减轻为SCLC预后不良因素^[3]^，本研究中虽然在生存分析部分未能得出体重减轻患者复发风险高的统计学差异，但体重减轻患者的NSE水平更高，仍提示体重下降与预后有一定关系。

治疗前后NSE及ProGRP的变化可反应疾病进程及对治疗的反应^[9]^。本研究中治疗有效的患者NSE及ProGRP水平较化疗前明显下降，二者的浓度差异在各疗效组有差异，提示肿瘤标志物变化程度可能有预测化疗效果的作用。

本研究以PFS为研究终点，复发类型按敏感、耐药及难治分组进行分析，发现随着三组复发类型肿瘤复发时间的缩短，治疗后ProGRP下降的程度逐渐减少，提示ProGRP在早期判断复发方面有一定参考作用。考虑到不同分期患者预后不同，按LD及ED分组，对患者进行生存分析。在单因素分析中，LD组化疗周期数 > 4周期的患者复发风险更小，在多因素分析中亦得到验证，提示对于局限期SCLC，足程治疗可能降低复发风险。两组中治疗前较高的NSE水平都使得复发风险增高，但均无统计学差异，考虑与样本量不足有关。ED组中疗前有较高的LDH水平者复发风险增高（*P*=0.036），提示LDH可能与SCLC的预后相关，与既往文献相符。经2个周期化疗后，LD组和ED组的ProGRP水平下降 > 70%者复发风险小（*P*=0.037, *P*=0.010），在多因素分析中进一步验证了ED组的结果（*P*=0.014），提示ProGRP可做为预测复发因素。既往研究^[10]^提示早放疗的远期疗效优于晚放疗，本研究中LD组行同步胸部放疗的患者复发风险比行序贯胸部放疗的患者低，但未达到统计学差异。ED组中远处转移部位数目是一项预后因素^[3]^，在本研究中有 > 2个远处转移病灶的患者复发风险更高（*P*=0.031），符合既往研究结果。

本项研究的不足之处在于为回顾性，样本量仍需进一步积累，以便得到更稳健的结果。总之，血清ProGRP、NSE及LDH在SCLC的分期、疗效评价及对复发风险评估方面有一定作用，ProGRP在预测复发方面更具优势，联合应用可在临床中发挥更好的作用。

## References

[1] Harmsma M, Schutte B, Ramaekers FC (2013). Serum markers in small cell lung cancer: opportunities for improvement. J Biochim Biophys Acta.

[2] Schneider J, Philipp M, Salewski L (2003). Pro-gastrin-releasing peptide (ProGRP) and neuron specific enolase (NSE) in therapy control of patients with small-cell lung cancer. J Clin Lab.

[3] Bremnes RM, Sundstrom S, Aasebo U (2003). The value of prognostic factors in small cell lung cancer: results from a randomised multicenter study with minimum 5 year follow-up. J Lung Cancer.

[4] Eisenhauer EA, Therasse P, Bogaerts J (2009). New response evaluation criteria in solid tumours: revised RECIST guideline (version 1.1). Eur J Cancer.

[5] Zhang WJ, Zhou ZM, Chen DF (2016). Clinical outcomes of extensive stage small cell lung cancer patients treated with intensive modified radiotherapy. Zhonghua Fang She Zhong Liu Xue Za Zhi.

[6] Bonner JA, Sloan JA, Rowland KM (2000). Significance of neuron-specific enolase levels before and during therapy for small cell lung cancer. J Clin Cancer Res.

[7] Miyake Y, Kodama T, Yamaguchi K (1994). Pro-gastrin-releasing peptide(31-98) is a specific tumor marker in patients with small cell lung carcinoma. J Cancer Res.

[8] Komatsu T, Oizumi Y, Kunieda E (2011). Definitive chemoradiotherapy of limited-disease small cell lung cancer: Retrospective analysis of new predictive factors affecting treatment results. J Oncol Lett.

[9] Petrovic M, Bukumiric Z, Zdravkovic V (2014). The prognostic significance of the circulating neuroendocrine markers chromogranin A, pro-gastrin-releasing peptide, and neuron-specific enolase in patients with small-cell lung cancer. J Med Oncol.

[10] Socinski MA, Bogart JA (2007). Limited-stage small-cell lung cancer: the current status of combined-modality therapy. J Clin Oncol.

